# Cross-Linking Mast Cell Specific Gangliosides Stimulates the Release of Newly Formed Lipid Mediators and Newly Synthesized Cytokines

**DOI:** 10.1155/2016/9160540

**Published:** 2016-08-08

**Authors:** Edismauro Garcia Freitas Filho, Elaine Zayas Marcelino da Silva, Camila Ziliotto Zanotto, Constance Oliver, Maria Célia Jamur

**Affiliations:** ^1^Department of Cell and Molecular Biology and Pathogenic Bioagents, Ribeirão Preto Medical School, University of São Paulo, Avenida Bandeirantes 3900, 14049-900 Ribeirão Preto, SP, Brazil; ^2^Department of Pharmacology, Ribeirão Preto Medical School, University of São Paulo, Avenida Bandeirantes 3900, 14049-900 Ribeirão Preto, SP, Brazil

## Abstract

Mast cells are immunoregulatory cells that participate in inflammatory processes. Cross-linking mast cell specific GD1b derived gangliosides by mAbAA4 results in partial activation of mast cells without the release of preformed mediators. The present study examines the release of newly formed and newly synthesized mediators following ganglioside cross-linking. Cross-linking the gangliosides with mAbAA4 released the newly formed lipid mediators, prostaglandins D_2 _and E_2_, without release of leukotrienes B_4_ and C_4_. The effect of cross-linking these gangliosides on the activation of enzymes in the arachidonate cascade was then investigated. Ganglioside cross-linking resulted in phosphorylation of cytosolic phospholipase A_2_ and increased expression of cyclooxygenase-2. Translocation of 5-lipoxygenase from the cytosol to the nucleus was not induced by ganglioside cross-linking. Cross-linking of GD1b derived gangliosides also resulted in the release of the newly synthesized mediators, interleukin-4, interleukin-6, and TNF-*α*. The effect of cross-linking the gangliosides on the MAP kinase pathway was then investigated. Cross-linking the gangliosides induced the phosphorylation of ERK1/2, JNK1/2, and p38 as well as activating both NF*κ*B and NFAT in a Syk-dependent manner. Therefore, cross-linking the mast cell specific GD1b derived gangliosides results in the activation of signaling pathways that culminate with the release of newly formed and newly synthesized mediators.

## 1. Introduction

Gangliosides are sialic acid containing glycosphingolipids that are present in the outer leaflet of the plasma membrane as well as in the membranes of some organelles [1, 2]. Gangliosides play a role in diverse physiological processes including growth, differentiation, cell-cell interactions, and cell signaling. They are also involved in many pathological processes, acting as receptors for viruses and toxins, and are implicated in tumor progression, atherosclerosis, and neurodegenerative disorders [3].

Gangliosides are present on the surface of mast cells and are critical for mast cell function [1]. Mast cells are multifunctional immune cells that participate in various biological events, such as inflammation and allergy. Mast cell functions are directly related to their activation and subsequent release of biologically active mediators [4, 5]. Mast cell activation via the high affinity IgE receptor (Fc*ε*RI) is the best characterized form of activation. It occurs when multivalent antigens cross-link antigen-specific immunoglobulin E (IgE) bound to FcɛRI on the mast cell surface. Cross-linking Fc*ε*RI initiates a signal transduction cascade that is dependent on the tyrosine kinase Syk [6]. This activation results in the release of three classes of mediators: preformed mediators such as histamine, proteases, cytokines, and enzymes; newly formed lipid mediators which are comprised of prostaglandins (PG), leukotrienes (LT), and platelet activating factor; and newly synthesized mediators which include cytokines and chemokines [4, 7, 8].

Mast cell specific gangliosides derived from GD1b are present on the surface of rodent mast cells [9, 10]. Cross-linking the GD1b derived gangliosides with a ganglioside specific monoclonal antibody (mAbAA4) or its F(ab′)_2_ fragment results in partial activation of RBL-2H3 mast cells without degranulation or release of preformed mediators [11, 12]. Although cross-linking GD1b derived gangliosides activates mast cells, whether or not cross-linking these gangliosides stimulates release of newly formed and newly synthesized mediators has not been investigated. Therefore, it was of interest to determine if newly formed lipid mediators and newly synthesized mediators were released following ganglioside cross-linking and whether or not this release was Syk-dependent.

## 2. Materials and Methods

### 2.1. Cells

RBL-2H3 cells, a rat mast cell line [13], a Syk-negative variant of RBL-2H3 cells (C4A2) [14], the stable RBL-2H3 cell lines expressing NF*κ*B-GFP reporter (NF*κ*B2; [15]); NFAT-GFP reporter (VB9; [16]); and Syk-negative cell lines expressing NF*κ*B-GFP reporter (IC2; [6]); and NFAT-GFP reporter (IH10; [16]) were generously provided by Dr. Reuben P. Siraganian (National Institute of Dental and Craniofacial Research, National Institutes of Health, Bethesda, MD). Cells were grown as monolayers at 37°C in Dulbecco's modified Eagle's medium (DMEM) supplemented with 15% fetal calf serum, 0.434 mg/mL glutamine, and an antibiotic-antimycotic mixture containing 100 U/mL penicillin, 100 *μ*g/mL streptomycin, and 0.25 *μ*g/mL amphotericin B (all from Life Technologies, Gibco, Carlsbad, CA) in a humidified incubator with 5% CO_2_ in air. Transfected cells were selected with Geneticin (0.4 mg/mL) (Sigma-Aldrich; St. Louis, MO).

### 2.2. Antibodies

Mouse monoclonal antibody anti-rat GD1b derived gangliosides (mAbAA4) were purchased from BD Biosciences (San Jose, CA). Rabbit polyclonal antibody anti-human phospho-cPLA_2_; rabbit polyclonal antibody anti-human cPLA_2_; rabbit mAb anti-human phospho-ERK1/2; rabbit mAb anti-rat ERK1/2; rabbit mAb anti-human phospho-JNK1/2; rabbit polyclonal antibody anti-human JNK1/2; rabbit mAb anti-human phospho-p38; rabbit polyclonal antibody anti-human anti-p38, and rabbit polyclonal antibody anti-human *α*/*β*-tubulin were purchased from Cell Signaling Technology Inc. (Danvers, MA). Rabbit polyclonal antibody anti-rat cyclooxygenase-2 (COX-2), rabbit mAb anti 5-lipoxygenase (5-LO), and rabbit polyclonal antibody anti-mouse Lamin B1 were purchased from Abcam (Cambridge, MA). Donkey anti-rabbit IgG conjugated to horseradish peroxidase (HRP) (Jackson ImmunoResearch Laboratories Inc., West Grove, PA) was used as the secondary antibody.

### 2.3. Mast Cell Activation and Mediator Release

In order to cross-link the GD1b derived gangliosides, RBL-2H3 cells and C4A2 cells were incubated with mAbAA4 at various concentrations (1, 2.5, 5, or 10 *μ*g/mL) depending on the experiment. For stimulation via Fc*ε*RI, cells were sensitized overnight (ON) with mouse IgE anti-TNP ascites fluid (1 : 5,000 dilution) and then stimulated with 50 ng/mL of DNP_48_-HSA (Sigma-Aldrich) for 30 min or for 1 h and then rinsed and cultured for an additional 3 h, for the release of newly formed lipid mediators (PGD_2_, PGE_2_, LTB_4_, and LTC_4_). In order to examine the release of newly synthesized mediators (IL-4, IL-6, and TNF-*α*), cells were stimulated for 1 h, rinsed, and cultured for an additional 11 h. For Fc*ε*RI independent stimulation, cells were incubated with 0.1 *μ*g/mL of calcium ionophore A23187 (Sigma-Aldrich). PGD_2_, PGE_2_, LTB_4_, and LTC_4 _in culture supernatants were analyzed using EIA kits (Cayman Chemical, Ann Arbor, MI). IL-4, IL-6, and TNF-*α* in the culture supernatants were measured using ELISA kits (BD Biosciences) according to the manufacturer's instructions. Nonstimulated cells were used as controls.

### 2.4. NF*κ*B and NFAT Activation

NF*κ*B2 cells, VB9 cells, IC2 cells, and IH10 cells were incubated with mAbAA4, stimulated via Fc*ε*RI, or with calcium ionophore for 1 h (as described in Section 2.3), rinsed, and cultured for an additional 5 h (NF*κ*B activation) or 15 h (NFAT activation). Cells were analyzed by flow cytometry and the percent of GFP positive cells was determined using a Guava Easy Cyte Mini System and Cytosoft Blue software (Guava Technologies Inc., Hayward, CA).

### 2.5. Immunoblotting

Total cells lysates were obtained as previously described [17]. For some experiments, nuclear and cytosolic extracts were obtained as previously described [18]. The proteins were separated electrophoretically on 8% or 12% polyacrylamide gels and transferred to Hybond membranes (GE Healthcare Life Sciences, Marlborough, MA). After transfer, the membranes were blocked for 1 h at RT in TTBS (0.05 M Tris-HCl, 0.15 M NaCl, pH 7.5, and 0.05% Tween 20) containing 4% BSA (Sigma-Aldrich). After blocking, the membranes were incubated ON at 4°C with the primary antibodies diluted in TTBS. The membranes were then washed, incubated for 30 min with secondary antibody, and developed using enhanced chemiluminescence (ECL Kit; GE Healthcare). The images were obtained with ImageQuant LAS 4000 (GE Healthcare). Mean optical density of the target protein was determined using ImageJ software (NIH).

### 2.6. Statistical Analyses

Results were analyzed using GraphPad Prism (GraphPad Software, Inc., San Diego, CA). The results were expressed as mean ± SD and differences between experimental samples were assessed by one-way analysis of variance (ANOVA) with Bonferroni's post hoc test; *P* < 0.05 was considered statistical significant.

## 3. Results

### 3.1. Cross-Linking GD1b Derived Gangliosides with mAbAA4 Induced the Release of Newly Formed Lipid Mediators PGD_2_ and PGE_2_

**Figure 1:**
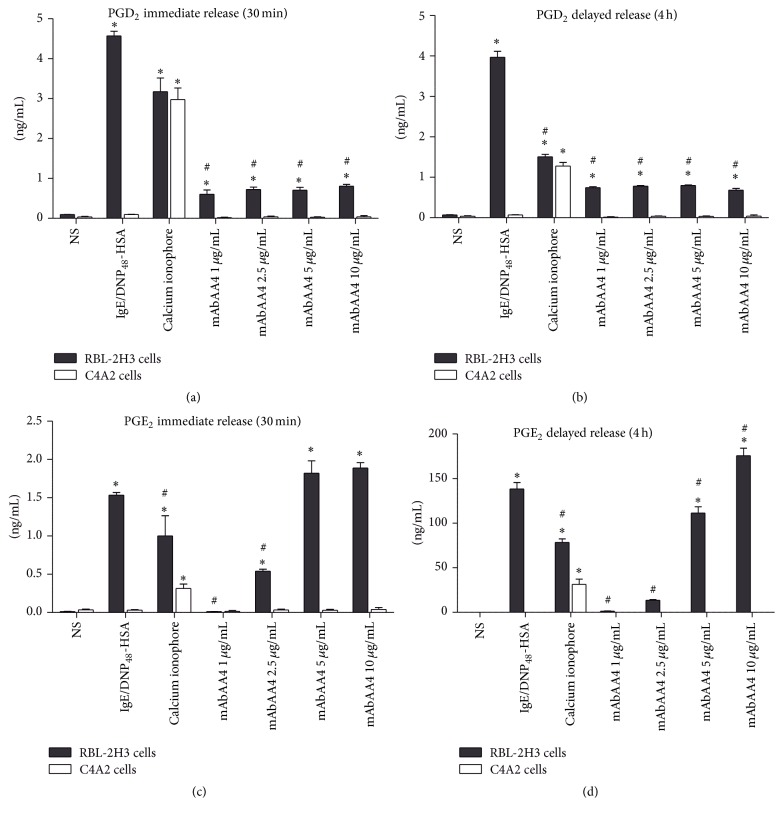
Cross-linking GD1b derived gangliosides by mAbAA4 induced Syk-dependent release of PGD_2_ and PGE_2_. For stimulation via Fc*ε*RI, RBL-2H3 cells and C4A2 Syk-negative cells were sensitized with IgE anti-TNP and stimulated with DNP_48_-HSA (50 ng/mL). For Fc*ε*RI independent stimulation, the cells were incubated with calcium ionophore (0.1 *μ*g/mL). To cross-link GD1b derived gangliosides, cells were incubated with mAbAA4 (1, 2.5, 5, and 10 *μ*g/mL). Nonstimulated (NS) cells were used as negative controls. Culture supernatants were collected after 30 min of incubation to evaluate immediate release (a and c) or cells were incubated for 1 h and rinsed and cultured for an additional 3 h and culture supernatants were collected to evaluate delayed release (b and d). PGD_2_ (a and b) and PGE_2_ (c and d) were measured in the culture supernatant by EIA. Data is expressed as the mean ± SD of three independent experiments. ^*∗*^ *P* < 0.05 between experimental samples and the nonstimulated (NS) cells. ^#^ *P* < 0.05 between experimental samples and Fc*ε*RI stimulated RBL-2H3 cells.

RBL-2H3 cells and C4A2 Syk-negative cells were incubated with mAbAA4 for either 30 min or 1 h and then rinsed and cultured for an additional 3 h to evaluate both immediate and delayed release of lipid mediators. The cross-linking of GD1b derived gangliosides by mAbAA4 induced both immediate and delayed release of PGD_2_ (Figures 1(a) and 1(b)) and PGE_2_ (Figures 1(c) and 1(d)) by RBL-2H3 cells, but not by Syk-negative C4A2 cells (Figures 1(a)–1(d)). Furthermore, the amount of PGE_2_ released following ganglioside cross-linking was higher when compared to that found after Fc*ε*RI stimulation. Interestingly, cross-linking GD1b derived gangliosides did not induce the release of the LT, LTB_4_, and LTC_4_ (see Supplementary Figures 1(A) and 1(B) in Supplementary Material available online at http://dx.doi.org/10.1155/2016/9160540).

### 3.2. Cross-Linking GD1b Derived Gangliosides with mAbAA4 Resulted in Phosphorylation of Cytosolic Phospholipase A_2_ (cPLA_2_) and Cyclooxygenase-2 (COX-2) Expression

**Figure 2:**
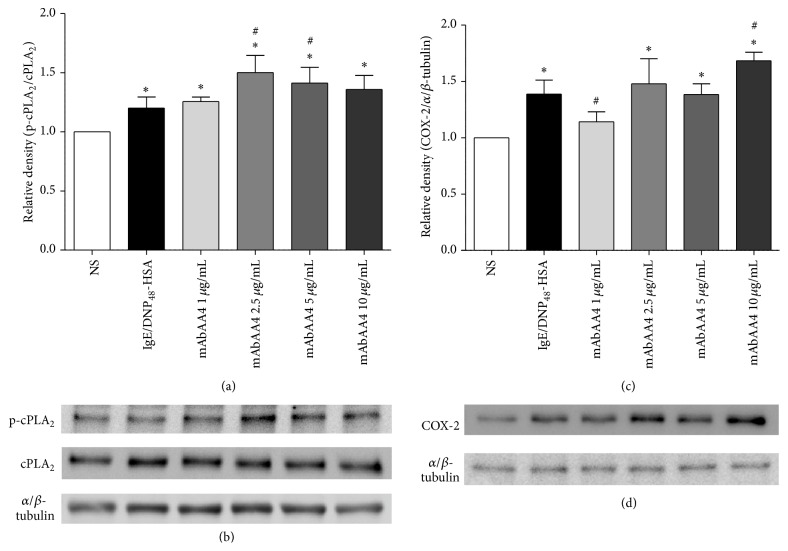
Cross-linking GD1b derived gangliosides by mAbAA4 increased cPLA_2_ phosphorylation and induced COX-2 expression. Either RBL-2H3 mast cells were stimulated via Fc*ε*RI, by sensitizing the cells with IgE anti-TNP and stimulating with DNP_48_-HSA (50 ng/mL), or cells were incubated with mAbAA4 (1, 2.5, 5, and 10 *μ*g/mL) for 5 min (cPLA_2_ phosphorylation) or for 1 h and then rinsed and cultured for an additional 3 h (COX-2 expression). Total cell lysates were immunoblotted with antibodies against p-cPLA_2_, cPLA_2_, COX-2, and *α*/*β*-tubulin (housekeeping protein) and the mean optical density of the bands was determined. Densitometry of the changes in expression and phosphorylation of proteins were corrected for *α*/*β*-tubulin. Data were expressed as the fold of nonstimulated (NS) cells. (a) Ratio of phosphorylated cPLA_2_/total cPLA_2_; (b) a representative blot from (a); (c) ratio of COX-2/*α*/*β*-tubulin; (d) a representative blot from (c). Data is expressed as the mean ± SD of three independent experiments. ^*∗*^ *P* < 0.05 between experimental samples and the nonstimulated (NS) cells. ^#^ *P* < 0.05 between experimental samples and Fc*ε*RI stimulated cells.

Cross-linking GD1b derived gangliosides resulted in the release of PGs but not LTs. Therefore, cPLA_2_ phosphorylation and induction of COX-2 expression, which are required for PG generation, were investigated. An increase in cPLA_2_ phosphorylation was observed after incubation of RBL-2H3 cells with mAbAA4 for 5 min and the levels of cPLA_2_ phosphorylation were higher than those observed in cells stimulated via Fc*ε*RI (Figures 2(a) and 2(b)). COX-2 expression was also induced in cells incubated with mAbAA4 for 1 h and rinsed and cultured for an additional 3 h (Figures 2(c) and 2(d)). In contrast, translocation of 5-LO from the cytosol to the nucleus, a requirement for LT generation, was not induced by ganglioside cross-linking (Supplementary Figures 1(C)–1(F)). Therefore, the cross-linking of GD1b derived gangliosides specifically induces the activation of the arachidonic pathway responsible for PG generation in mast cells.

### 3.3. Cross-Linking GD1b Derived Gangliosides with mAbAA4 Induced the Release of Newly Synthesized Cytokines

**Figure 3:**
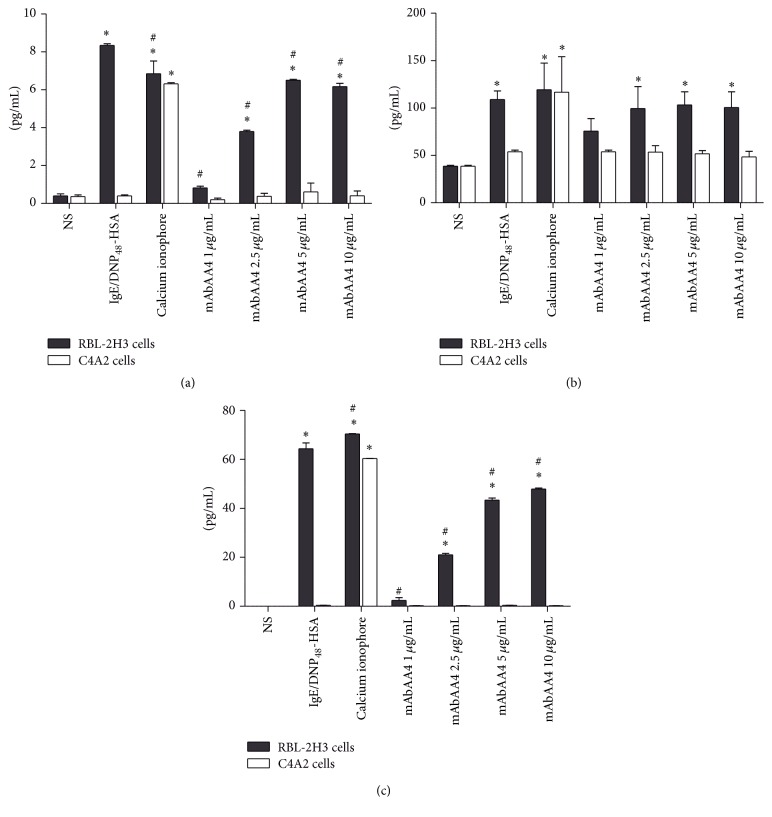
Cross-linking GD1b derived gangliosides with mAbAA4 induced the release of IL-4, IL-6, and TNF-*α* in a Syk-dependent manner. For stimulation via Fc*ε*RI, RBL-2H3 cells and C4A2 Syk-negative cells were sensitized with IgE anti-TNP and stimulated with DNP_48_-HSA (50 ng/mL). For Fc*ε*RI independent stimulation, the cells were incubated with calcium ionophore (0.1 *μ*g/mL). To cross-link GD1b derived gangliosides, cells were incubated with mAbAA4 (1, 2.5, 5, and 10 *μ*g/mL). Nonstimulated (NS) cells were used as negative controls. Culture supernatants were collected 1 h after stimulation and the cells were rinsed and cultured for an additional 11 h to evaluate cytokine release. IL-4 (a), IL-6 (b), and TNF-*α* (c) were measured in the culture supernatants by ELISA. Data is expressed as the mean ± SD of three independent experiments. ^*∗*^ *P* < 0.05 between experimental samples and the nonstimulated (NS) cells. ^#^ *P* < 0.05 between experimental samples and Fc*ε*RI stimulated RBL-2H3 cells.

Mast cell activation via Fc*ε*RI leads to transcription factor activation resulting in the production and release of cytokines [19]. Therefore, it was of interest to investigate whether cytokines are released after cross-linking GD1b derived gangliosides by mAbAA4. RBL-2H3 cells and Syk-negative C4A2 cells were incubated with mAbAA4 for 1 h and rinsed and cultured for an additional 11 h. Ganglioside cross-linking resulted in a Syk-dependent release of the newly synthesized mediators, interleukin-4 (IL-4) (Figure 3(a)), interleukin-6 (IL-6) (Figure 3(b)), and tumor necrosis factor-*α* (TNF-*α*) (Figure 3(c)). Interestingly, the amount of cytokines released after ganglioside cross-linking, with the exception of IL-6, was lower than that observed after stimulation via Fc*ε*RI.

#### 3.3.1. Cross-Linking GD1b Derived Gangliosides with mAbAA4 Induced MAP Kinase Phosphorylation

**Figure 4:**
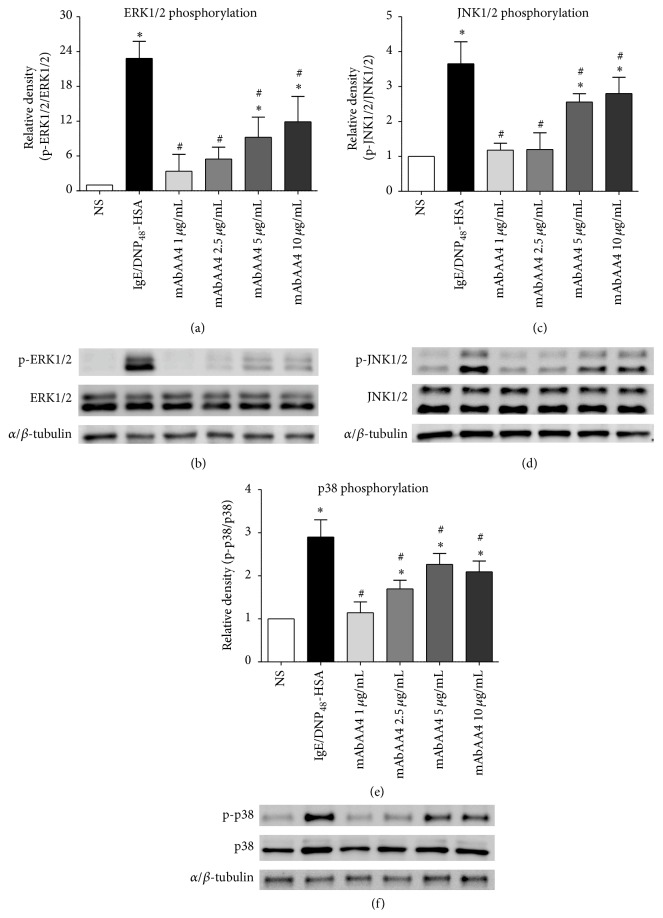
Cross-linking GD1b derived gangliosides with mAbAA4 induced MAP kinase phosphorylation in mast cells. RBL-2H3 cells were either stimulated via Fc*ε*RI, where cells were sensitized with IgE anti-TNP and stimulated with DNP_48_-HSA (50 ng/mL) or incubated with mAbAA4 (1, 2.5, 5, and 10 *μ*g/mL) for 10 min. Total cell lysates were immunoblotted with antibodies against phospho-ERK1/2 (p-ERK1/2), ERK1/2, phospho-JNK1/2 (p-JNK1/2), JNK1/2, phospho-p38 (p-p38), p38, and *α*/*β*-tubulin (housekeeping protein) and the mean optical density of the bands was determined. Densitometry of the changes in expression and phosphorylation of proteins were corrected for *α*/*β*-tubulin. Data is expressed as the fold of nonstimulated (NS) cells. (a) Ratio of phosphorylated ERK1/2/total ERK1/2; (b) a representative blot from (a); (c) ratio of phosphorylated JNK1/2/total JNK1/2; (d) a representative blot from (c); (e) ratio of phosphorylated p38/total p38; (f) a representative blot from (e). Data is expressed as the mean ± SD of three independent experiments. ^*∗*^ *P* < 0.05 between experimental samples and the nonstimulated (NS) cells. ^#^ *P* < 0.05 between experimental samples and Fc*ε*RI stimulated cells.

MAP kinases are involved in signaling pathways that lead to production of newly synthesized mediators [20]. Since incubation of mast cells with mAbAA4 resulted in the release of IL-4, IL-6, and TNF-*α*, it was of interest to investigate the degree of MAP kinase phosphorylation induced by cross-linking the GD1b derived gangliosides. When RBL-2H3 cells were incubated with mAbAA4 for 10 min, MAP kinases ERK1/2, JNK1/2, and p38 were phosphorylated (Figure 4). The degree of MAP kinase phosphorylation in mast cells incubated with mAbAA4 was less than that observed in cells stimulated via Fc*ε*RI, which agrees with the amount of cytokine released.

#### 3.3.2. Cross-Linking GD1b Derived Gangliosides with mAbAA4 Induced the Activation of Transcription Factors

**Figure 5:**
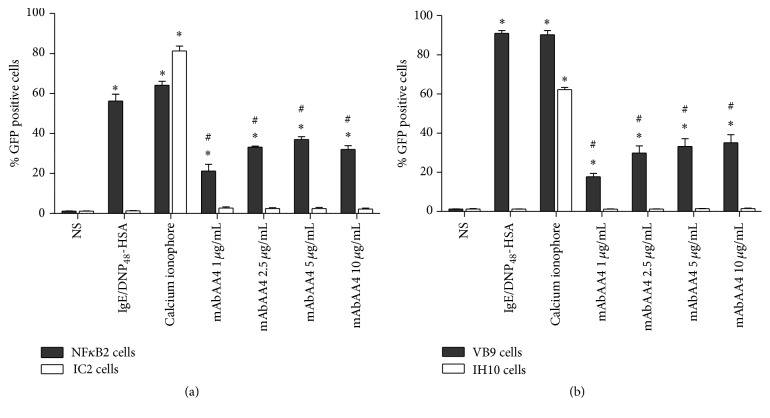
Cross-linking GD1b derived gangliosides by mAbAA4 induced the activation of the transcription factors NF*κ*B and NFAT. NF*κ*B2 cells, VB9 cells, IC2 cells, and IH10 cells were sensitized with IgE anti-TNP and stimulated with DNP_48_-HSA (50 ng/mL) for 1 h and rinsed and cultured for additional 5 h ((a); NF*κ*B) or 15 h ((b); NFAT). For Fc*ε*RI independent stimulation, the cells were incubated with calcium ionophore (0.1 *μ*g/mL). To cross-link GD1b derived gangliosides, cells were incubated with mAbAA4 (1, 2.5, 5, and 10 *μ*g/mL). GFP expression was analyzed by flow cytometry. Data is expressed as the mean ± SD of three independent experiments. ^*∗*^ *P* < 0.05 between experimental samples and the nonstimulated (NS) cells. ^#^ *P* < 0.05 between experimental samples and Fc*ε*RI stimulated cells.

Transcription factor activation is the ultimate requirement for the production of newly synthesized mediators [21]. Therefore, RBL-2H3 derived GFP reporter cell lines were used to assess NF*κ*B and NFAT activation. Cross-linking GD1b derived gangliosides by mAbAA4 induced activation of both NF*κ*B (Figure 5(a)) and NFAT (Figure 5(b)) in a Syk-dependent manner. However, transcription factor activation by ganglioside cross-linking was less prominent than that observed by stimulation via F*ε*RI.

## 4. Discussion

The present study demonstrates that cross-linking the mast cell specific GD1b derived gangliosides induces the release of newly formed and newly synthesized mediators. Furthermore, this release is Syk-dependent. However, previous investigations have demonstrated that mast cell activation by cross-linking these gangliosides does not induce the release of preformed mediators [11, 12]. Moreover, other studies have shown that cross-linking gangliosides can also activate a variety of cell types [22–26]. Antibodies to gangliosides have been shown to activate PKC and increase proliferation in lymphocytes [27, 28], stimulate calcium influx in oligodendrocytes [29], and induce leukocyte degranulation [30]. The molecular mechanisms by which cross-linking gangliosides can activate cells are poorly understood.

Eicosanoids (prostaglandins, thromboxane, leukotrienes, and lipoxins) are the most important lipid mediators generated by mast cells [31]. The results of the present study show that cross-linking GD1b derived gangliosides induces release of prostaglandins, but not leukotrienes. Incubation with mAbAA4 stimulated cPLA_2_ phosphorylation and incubation with mAbAA4 for extended periods of time increased COX-2 expression. The first step in eicosanoid generation is Ca^2+^-dependent phosphorylation of cPLA_2_ through the MAP kinase pathway. Phosphorylated cPLA_2_ translocates to cellular membranes, principally to the nuclear envelope, where arachidonic acid (AA) is released from membrane phospholipids by the action of cPLA_2_. AA is then metabolized either by COX-2 or CYP2E1 to produce PGs such as PGE_2_ or by 5-LO to produce LTs in concert with 5-lipoxygenase-activating protein (FLAP) on the nuclear envelope [32, 33]. Previous studies have shown that the immediate phase of PG generation (5–30 min) requires the action of constitutively expressed COX-1 and phosphorylation of cPLA_2_, while the delayed phase of PG generation (4–6 h) depends on the induced expression of COX-2 [34]. In addition, cross-linking GD1b derived gangliosides did not induce 5-LO translocation from the cytosol to the nuclear membrane. These results agree with the findings that ganglioside cross-linking induces release of prostaglandins, but not leukotrienes, and indicate that ganglioside cross-linking selectively stimulates the eicosanoid biosynthetic pathway to induce PG generation.

Cross-linking GD1b derived gangliosides induces the release of the newly synthesized mediators IL-4, IL-6, and TNF-*α*. In mast cells, newly synthesized mediator expression depends on the activation of signaling pathways that ultimately leads to transcription factor activation [21]. These events culminate with cytokine production and release and can occur even in the absence of mast cell degranulation [16]. A variety of studies investigating Fc*ε*RI independent mast cell activation also revealed that release of proinflammatory mediators can occur in the absence of degranulation [35–37]. The production of newly synthesized mast cell mediators following Fc*ε*RI activation relies on MAP kinase signaling pathways as well as on the activation of the transcription factors NF*κ*B and NFAT.

The MAP kinase signaling pathway participates in activation, differentiation, proliferation, and migration of mast cells. Cross-linking GD1b derived gangliosides results in ERK1/2, JNK1/2, and p38 MAP kinase phosphorylation. ERK1/2 is an essential signal in the production of the newly synthesized mediators IL-5, IL-3, IL-13, and TNF-*α* in mast cells [38]. JNK1/2 is responsible, at least partially, for the expression and production of several cytokines, including IL-6 and TNF-*α* in mast cells [39]. Additionally, activation of p38 MAP kinase was shown to stimulate IL-4 production in bone marrow derived mast cells [40]. When mast cells are stimulated via Fc*ε*RI, the transcription factors NF*κ*B and NFAT are translocated to the nucleus and initiate the transcription of genes for proinflammatory and regulatory cytokines. This results in the expression and release of cytokines [19, 41, 42]. Cross-linking GD1b derived gangliosides by mAbAA4 activates the transcription factors NF*κ*B and NFAT. However, the degree of activation by ganglioside cross-linking was less than that observed by stimulation via Fc*ε*RI. This reduction in activation is expected since the degree of MAP kinase phosphorylation after ganglioside cross-linking was less than that observed in mast cells stimulated via Fc*ε*RI. Similar results have been reported for phosphorylation of Lyn, Syk, PLC*γ*1, and the *β*- and *γ*-subunits of Fc*ε*RI [12].

The lower phosphorylation of MAP kinase resulted in a reduction in NF*κ*B and NFAT activation leading to a decrease in IL-4 and TNF-*α* release. In Fc*ε*RI stimulated mast cells, activation of NF*κ*B depends on PKC activation [43]. On the other hand, NFAT is activated by calcineurin induced dephosphorylation, a Ca^2+^-calmodulin dependent serine/threonine phosphatase that is activated by an increase in intracellular calcium [44, 45]. mAbAA4 binding to RBL-2H3 mast cells results in a modest increase in intracellular calcium as well as in a partial redistribution of PKC [11], which could explain the reduced activation of NF*κ*B and NFAT seen in the present study. Additionally, cross-linking GD1b derived gangliosides in Syk-negative cells did not stimulate the release of either newly formed or newly synthesized mediators. This is in agreement with previous studies that have shown that the inhibition or the lack of Syk results in the failure of mast cells to produce and release any mediators [46, 47]. Syk-negative mast cells are also unable to activate NF*κ*B and NFAT in response to Fc*ε*RI activation [6, 16].

The exact mechanism by which cross-linking the GD1b derived gangliosides causes the various effects observed both previously and in this study is still unknown. Several intracellular signals induced by mAbAA4 binding are very similar to those induced by Fc*ε*RI activation. Binding of mAbAA4 to mast cells is known to stimulate protein tyrosine phosphorylation, including phosphorylation of Lyn, Syk, PLC*γ*1, and the *β*- and *γ*-subunits of Fc*ε*RI. However, the rate of phosphorylation of Lyn, Syk, and PLC*γ*1 was slower with ganglioside cross-linking than with Fc*ε*RI stimulation [12]. In addition to these effects of mAbAA4, preincubation of RBL-2H3 cells with mAbAA4 selectively inhibits the degranulation induced by Fc*ε*RI stimulation at a very early step of upstream receptor tyrosine phosphorylation. This inhibition is unrelated to mAbAA4 blocking IgE-binding to the cells [48, 49]. Moreover, the GD1b derived gangliosides coimmunoprecipitate with Fc*ε*RI [48] as well as with the tyrosine kinase Lyn [49]. Oliver et al. [50] demonstrated that in RBL-2H3 cells stimulated via Fc*ε*RI, the gangliosides and Fc*ε*RI are internalized together and follow the same intracellular endocytic pathway suggesting that the GD1b derived gangliosides are involved in the organization of the signaling complex.

## 5. Conclusions

The present study has demonstrated that cross-linking the GD1b derived gangliosides stimulates the release of newly formed and newly synthesized mediators. Although these gangliosides are intimately associated with Fc*ε*RI, the ability of the gangliosides to activate mast cells is not dependent on Fc*ε*RI cross-linking. The present study helps to explain the extremely broad spectrum of potential mechanisms by which mast cells might act in suppressing, amplifying, and modulating the non-Fc*ε*RI mediated immune responses. Furthermore, an understanding of the role of gangliosides in mast cell activation may lead to new therapeutic targets for allergic and inflammatory processes.

## Supplementary Material

Supplementary Fig. 1. Cross-linking GD1b derived gangliosides by mAbAA4 did not induce the release of LTB4 or LTC4 or the translocation of 5-LO. In order to evaluate leukotriene release, RBL-2H3 cells and C4A2 Syk-negative cells were sensitized with IgE anti-TNP and stimulated with DNP48-HSA (50 ng/mL) for stimulation via Fc epsilon RI. For Fc epsilon RI independent stimulation, the cells were incubated with calcium ionophore (0.1 µg/mL). To cross-link GD1b derived gangliosides, cells were incubated with mAbAA4 (1, 2.5, 5, and 10 µg/mL). Non-stimulated (NS) cells were used as negative controls. Culture supernatants were collected after 30 min to evaluate LT release. LTB4 (A) and LTC4 (B) were measured in the culture supernatants by EIA. To examine 5-LO translocation, RBL-2H3 cells were either stimulated via FcεRI, where cells were sensitized with IgE anti-TNP and stimulated with DNP48-HSA (50 ng/mL), or incubated with mAbAA4 (1, 2.5, 5, and 10 µg/mL) for 5 min. Cytosolic and nuclear lysates were immunoblotted with antibodies against 5-LO, α/β-tubulin, and Lamin B1 and the mean optical density of the bands was determined. Data were expressed as the fold of non-stimulated (NS) cells. (C) ratio of cytosolic 5-LO (C-5-LO)/α/β-tubulin (housekeeping protein from the cytosolic fraction); (D) a representative blot from C; (E) ratio of nuclear 5-LO (N-5-LO)/Lamin B1 (housekeeping protein from the nuclear fraction); (F) a representative blot from E. Data is expressed as the mean ± SD of three independent experiments. ∗P<0.05 between experimental samples and the non-stimulated (NS) cells. #P<0.05 between experimental samples and Fc*ε*RI stimulated cells.
