# Supplementary material for: Cross-Linking Mast Cell Specific Gangliosides Stimulates the Release of Newly Formed Lipid Mediators and Newly Synthesized Cytokines
Source: Mediators Inflamm. 2016 Aug 8;2016:9160540. doi: 10.1155/2016/9160540 (PMC4992799; doi:10.1155/2016/9160540)
Supplement: Supplementary file 1 — Supplementary Fig. 1. Cross-linking GD1b derived gangliosides by mAbAA4 did not induce the release of LTB4 or LTC4 or the translocation of 5-LO. In order to evaluate leukotriene release, RBL-2H3 cells and C4A2 Syk-negative cells were sensitized with IgE anti-TNP and stimulated with DNP48-HSA (50 ng/mL) for stimulation via Fc epsilon RI. For Fc epsilon RI independent stimulation, the cells were incubated with calcium ionophore (0.1 µg/mL). To cross-link GD1b derived gangliosides, cells were incubated with mAbAA4 (1, 2.5, 5, and 10 µg/mL). Non-stimulated (NS) cells were used as negative controls. Culture supernatants were collected after 30 min to evaluate LT release. LTB4 (A) and LTC4 (B) were measured in the culture supernatants by EIA. To examine 5-LO translocation, RBL-2H3 cells were either stimulated via FcεRI, where cells were sensitized with IgE anti-TNP and stimulated with DNP48-HSA (50 ng/mL), or incubated with mAbAA4 (1, 2.5, 5, and 10 µg/mL) for 5 min. Cytosolic and nuclear lysates were immunoblotted with antibodies against 5-LO, α/β-tubulin, and Lamin B1 and the mean optical density of the bands was determined. Data were expressed as the fold of non-stimulated (NS) cells. (C) ratio of cytosolic 5-LO (C-5-LO)/α/β-tubulin (housekeeping protein from the cytosolic fraction); (D) a representative blot from C; (E) ratio of nuclear 5-LO (N-5-LO)/Lamin B1 (housekeeping protein from the nuclear fraction); (F) a representative blot from E. Data is expressed as the mean ± SD of three independent experiments. ∗P<0.05 between experimental samples and the non-stimulated (NS) cells. #P<0.05 between experimental samples and FcεRI stimulated cells. [file 9160540.f1.pdf]

Supplementary Material

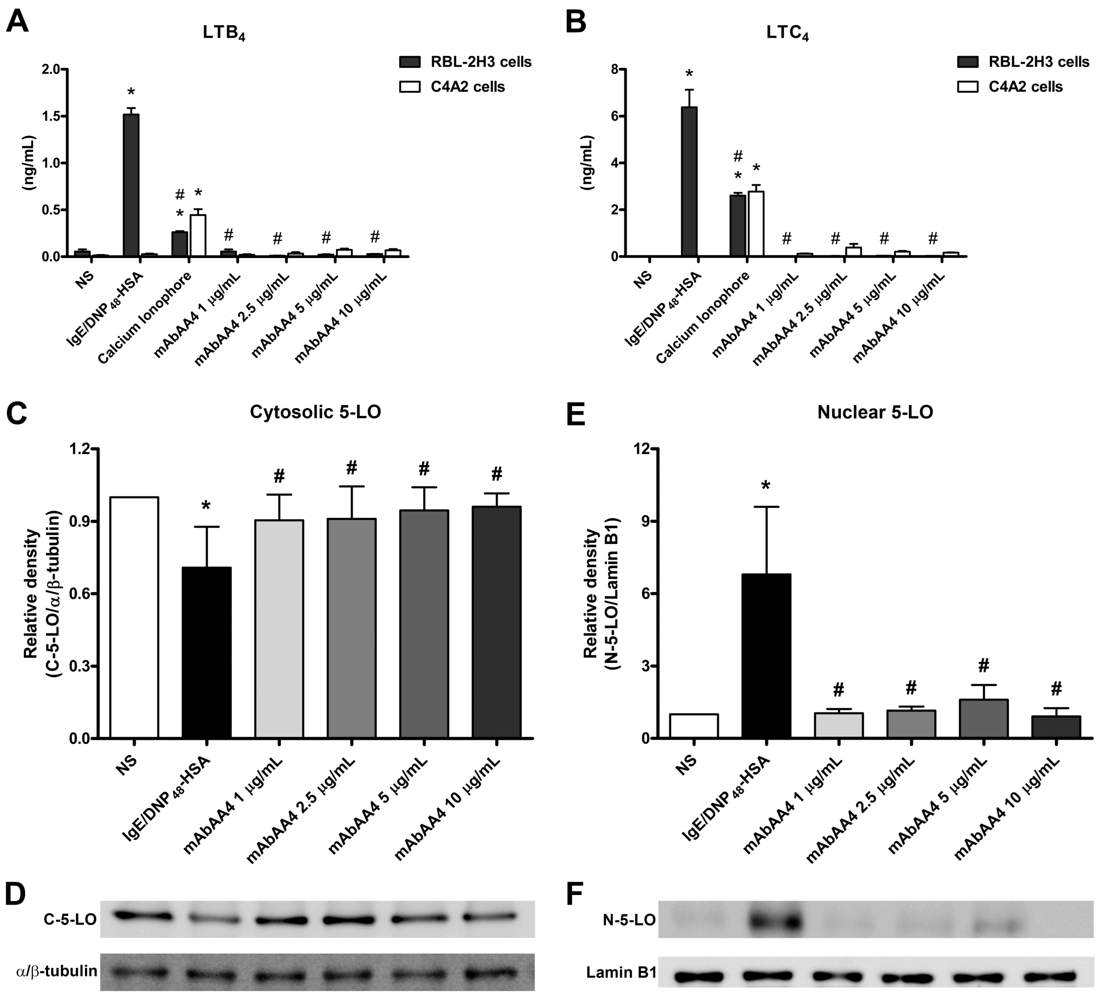

Supplementary Fig. 1. Cross-linking GD1b derived gangliosides by mAbAA4 did not induce the release of LTB_4 or LTC_4 or the translocation of 5-LO. In order to evaluate leukotriene release, RBL-2H3 cells and C4A2 Syk-negative cells were sensitized with IgE anti-TNP and stimulated with DNP_{48}-HSA (50 ng/mL) for stimulation via FcεRI. For FcεRI independent stimulation, the cells were incubated with calcium ionophore (0.1 μg/mL). To cross-link GD1b derived gangliosides, cells were incubated with mAbAA4 (1, 2.5, 5, and 10 μg/mL). Non-stimulated (NS) cells were used as negative controls. Culture supernatants were collected after 30 min to evaluate LT release. LTB_4 (A) and LTC_4 (B) were measured in the culture

supernatants by EIA. To examine 5-LO translocation, RBL-2H3 cells were either stimulated via FcεRI, where cells were sensitized with IgE anti-TNP and stimulated with DNP_{48}-HSA (50 ng/mL), or incubated with mAbAA4 (1, 2.5, 5, and 10 μg/mL) for 5 min. Cytosolic and nuclear lysates were immunoblotted with antibodies against 5-LO, α/β-tubulin, and Lamin B1 and the mean optical density of the bands was determined. Data were expressed as the fold of non-stimulated (NS) cells. (C) ratio of cytosolic 5-LO (C-5-LO)/α/ β-tubulin (housekeeping protein from the cytosolic fraction); (D) a representative blot from C; (E) ratio of nuclear 5-LO (N-5-LO)/Lamin B1 (housekeeping protein from the nuclear fraction); (F) a representative blot from E. Data is expressed as the mean ± SD of three independent experiments. *$P<0.05$ between experimental samples and the non-stimulated (NS) cells. $^{\#}P<0.05$ between experimental samples and FcεRI stimulated cells.
